# Do sports develop leadership? The impact of college varsity sports on leadership capacity and self-efficacy

**DOI:** 10.3389/fspor.2025.1691139

**Published:** 2025-11-18

**Authors:** Benjamin P. Correia-Harker, Lindsey M. Clark, Jacob J. Capin

**Affiliations:** 1 Department of Educational Policy and Leadership, Marquette University, Milwaukee, WI, United States; 2 Life After Sport Trajectories Lab, Department of Physical Therapy, College of Health Sciences, Marquette University, Milwaukee, WI, United States; 3 Clinical and Translational Institute of Southeast Wisconsin, Medical College of Wisconsin, Milwaukee, WI, United States

**Keywords:** athlete, competitive sport, leadership, leadership capacity, leadership efficacy, college athlete

## Abstract

**Introduction:**

Effective leadership in sports is associated with better team functioning, physical and psychosocial wellbeing, and reduced incidence of severe injuries. In spite of widespread anecdotal support, few studies have empirically evaluated leadership development in athletes. The purpose of this study was to investigate socially responsible leadership capacity and self-efficacy in college varsity athletes and determine the impact of competitive sports participation on leadership capacity and self-efficacy. We hypothesized that college varsity athletes would (1) exhibit greater leadership capacity and self-efficacy than nonathletes and (2) demonstrate greater growth in leadership capacity and self-efficacy than nonathletes.

**Methods:**

The Multi-Institutional Study of Leadership survey was distributed to a representative sample of United States college students in 2018. The leadership outcomes of college varsity (intercollegiate) athletes and nonathletes were compared using the Socially Responsible Leadership Scale and Leader Self-Efficacy Scale. The Socially Responsible Leadership Scale has an omnibus score and six subscales: self-awareness, personal responsibility, integrity, collaboration, open-mindedness, and civic engagement. Student’s t-tests were used to compare scores on the Socially Responsible Leadership Scale and Leader Self-Efficacy Scale between athletes and nonathletes. Hierarchical multiple linear regression analyses were conducted for each leadership outcome, both with and without leadership high-impact practices.

**Results:**

The study contained 50,653 participants, including 8,447 college varsity athletes (age 20.3 ± 1.5 years, 56.2% women) and 42,206 nonathletes (age 20.2 ± 1.5 years, 65.9% women). While athletes reported statistically higher levels of self-awareness and leader self-efficacy but lower integrity and open-mindedness compared with their nonathlete peers, all comparisons had trivial effect sizes (all Cohen's d < 0.02; range: −0.067 to 0.159). There were no differences between athletes and nonathletes in terms of personal responsibility, collaboration, civic engagement, and the omnibus score of the Socially Responsible Leadership Scale. In regression models with and without leadership high-impact practices, athlete status explained only 0.1% or less of variance in each leadership outcome.

**Discussion:**

The findings from this large sample of college athletes and nonathletes (*n* = 50,653) challenge widely held notions regarding sports and leadership, suggesting that competitive sports alone may not help people develop leadership capacity or self-efficacy. While sports provide opportunities for people to engage in leadership high-impact practices, athletes may benefit from additional resources to develop leadership skills inside and outside of sports.

## 1 Introduction

Participation in organized sports has many benefits and can positively impact athletes physiologically (1), mentally (2–5), and socially (5, 6). In addition to many physiological benefits, such as greater aerobic capacity and strength, the regular exercise associated with sports participation leads to higher self-efficacy (7) and self-esteem (8). A recent systematic review found that sports at the community and elite levels improved psychological wellbeing (e.g., self-esteem and life satisfaction) and social outcomes (e.g., self-control, prosocial behavior, interpersonal communication, and belonging) (5). The many benefits of sports and the common knowledge of how sports enhance physiological and psychosocial wellbeing lead many to believe that sports participation promotes leadership development, which is an aspect of psychosocial wellbeing and development (9). Moreover, sports are often proposed and touted in the media as a means to enhance leadership skills in athletes (10). Media reports also indicate that a high percentage of leaders—particularly women CEOs—participate in competitive sports (11, 12).

While numerous studies have investigated the impact of coaching leadership styles on athlete experiences (13–17) and injury rates (18), few have empirically evaluated leadership development in athletes (19, 20). Effective leadership in a sports setting is associated with better team functioning and efficacy (21, 22), physical and psychosocial health and wellbeing (21, 23), and reduced incidence of severe injuries (18). The National Collegiate Athletic Association promotes leadership development among its collegiate athletes (10). A recent qualitative study found that former competitive athletes noted many benefits from their lived experiences, including the leadership skills they gained from high-level sports (24). In contrast, a longitudinal study of 2,109 college students surveyed between fall 2006 and spring 2010 found, ironically, that intercollegiate athletic participation did not promote leadership development and that team sport athletes exhibited lower socially responsible leadership than nonathletes (20). Dugan et al. explored leadership development among students involved in intramural and club sports, finding that baseline leadership scores and high-impact leadership practices (e.g., sociocultural conversations with peers, community service, and mentoring relationships) (25)—which are common practices within many team sports—were key factors associated with leadership capacity and efficacy (26). More recent comparative studies of leadership development in athletes vs. nonathletes—and the factors associated with leadership development in athletes—are lacking.

Determining the impact of sports on leadership capacity and self-efficacy could influence sports policies, athlete development programs, and coaching and sports psychology/counseling. While there are numerous definitions of and theories on leadership used in the literature and practice (e.g., transformational, servant, and situational) (27), socially responsible leadership, as conceived in the Social Change Model of Leadership Development (SCM) approach, is one of the most widely used theories for leadership development in higher education (28). Because of its prominence, its alignment with the civic aims of higher education, and its emphasis on both individual and group capacities (29), we used the Socially Responsible Leadership Scale (SRLS) to measure leadership capacity in accordance with the SCM (30). In addition, both types of scholarship—theoretical and empirical—identify leadership efficacy as a meaningful predictor of leadership capacity (27, 31–33); therefore, leadership efficacy was also included as an outcome of interest in this study.

The purpose of this study was to investigate socially responsible leadership capacity and self-efficacy in college varsity athletes and determine the impact of college varsity sports participation on leadership capacity and self-efficacy. Specifically, we aimed to (1) compare leadership capacity and self-efficacy in college varsity athletes vs. nonathletes and (2) determine the impact of college varsity sports on leadership capacity and self-efficacy while controlling for baseline factors with and without leadership high-impact practices. According to widely held beliefs within sports, media reports (10–12), and qualitative research (24), our hypotheses were that (1) athletes would exhibit greater leadership capacity and self-efficacy than nonathletes and (2) athletes would demonstrate greater growth in leadership capacity and self-efficacy than nonathletes.

## 2 Methods

### 2.1 Study design

Data were collected using the Multi-Institutional Study of Leadership (MSL), an instrument used to examine leadership and leadership development in higher education. The MSL measures demographic factors, collegiate experiences, and leadership and leadership-related outcomes. The MSL has been administered every 3 years, but since the most recent dataset was prepared and made available only from the year 2021, which represented the height of the COVID-19 pandemic, we used the 2018 dataset so that the results would not reflect the challenges that presented during the pandemic.

The MSL is a cross-sectional survey that employs a quasi pretest design in which students respond after reflecting upon their time prior to college. This process is used for a few key reasons. First, the conceptual framework guiding the study is Astin's (34) inputs–environments–outputs model, which posits that to best understand the impact of an experience, researchers must first understand the circumstances and state of one entering an experience in order to fully understand how the subjects change as a result of it (34). A quasi pretest remains constant for precollege mindsets and enables the measurement of outcome growth from the collegiate athletic experience. Second, the quasi pretest is used in lieu of a true longitudinal design because of feasibility and potential constraints of accuracy. Given the cognitive shifts that collegians may experience regarding the psychological constructs related to, and the abilities pertaining to, leadership and perceptions thereof, reflective pre-posttests may be more accurate measures of change than true prepost designs (35). As conceptions of leadership and perceptions of one's own engagement in leadership can shift over time, the quasi pre-posttest design may be more accurate. This design was chosen over a longitudinal pretest because of its feasibility and to account for cognitive shifts in how students conceptualize leadership. However, it relies on participants' accurate recall of their precollege states, which is a limitation.

### 2.2 Leadership surveys and psychometric properties

**Figure 1:**
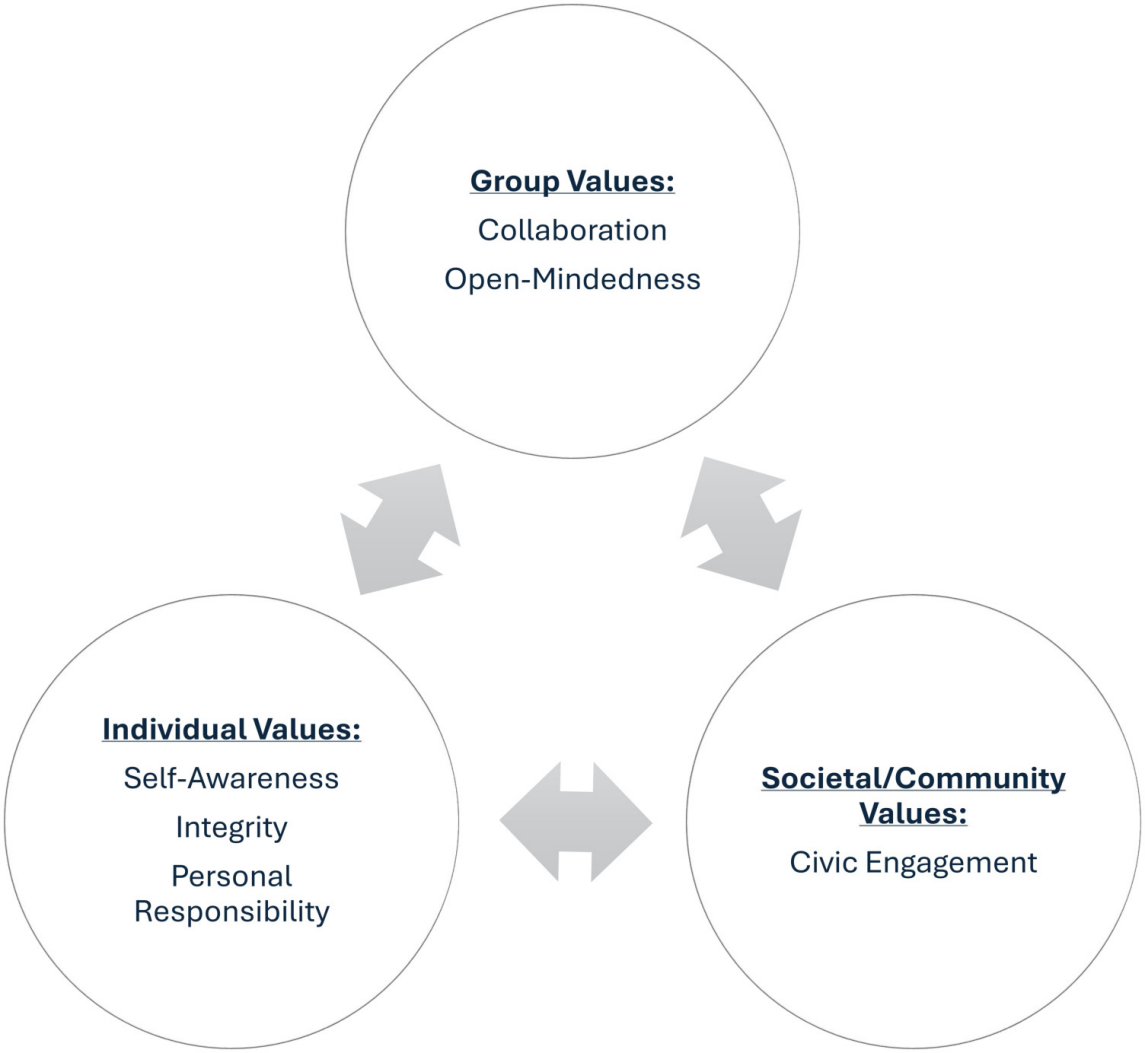
The Social Change Model of Leadership Development (SCM) adapted from the Higher Education Research Institute (1996) (29) with adjusted value labels aligning with the Socially Responsible Leadership Scale (SRLS). Used with permission from the National Clearinghouse for Leadership Program. Note that the terminology used in the present study reflects recently revised construct labels that resonate with a broader audience. Synonymous terminology that was previously used includes controversy with civility (i.e., open-mindedness), citizenship (i.e., civic engagement), consciousness of self (i.e., self-awareness), congruence (i.e., integrity), and commitment (i.e., personal responsibility).

The outcome variables for this study are the Socially Responsible Leadership Scale (SRLS) (29, 30) and the Leader Self-Efficacy (LSE) scale (36, 37). Considered a form of gauging leadership capacity, the SRLS measures leadership as conceptualized by the Social Change Model of Leadership Development (SCM) (29, 30). Although leadership can be conceptualized in many ways, the SRLS aligns well with modern conceptions of leadership (27), reflects the democratic aims of higher education (38), and is most commonly used in higher education in the United States (28). The SRLS consists of 34 items comprising 6 subscales, reflecting the SCM values of self-awareness, personal responsibility, integrity, collaboration, open-mindedness, and civic engagement (Figure 1) (39). [Common purpose is included in the SCM but is excluded from the SRLS because it is not sufficiently divergent from collaboration (39)]. The response options for the SRLS items range from 1 (*strongly disagree*) to 5 (*strongly agree*).

LSE represents the internal belief that one has in one’s ability to engage in leadership (36, 37). Scholars theoretically and empirically associate LSE with leadership capacity and engagement (27, 31, 32). LSE is measured using a 4-point scale, with 1 being *not at all confident* and 4 being *very confident.*

### 2.3 Participants

The MSL was administered online to a random sample of undergraduate students at institutions across the United States. Higher education institutions voluntarily enrolled in the study and provided a random sample of 4,000 students or a census sample if their enrollment amounted to fewer than 4,000 students. Participants provided written informed consent to participate in this IRB-approved study (IRB Project Number 2,328 from Loyola University Chicago). The MSL was administered to 256,289 undergraduate students between January and May 2018 with a 29% response rate. The sample is representative of higher education institutions based on size, affiliation, and classification. In the present study, age was restricted to 18–25 years, given that most college athletes fall in this age range. The final analytic sample size after applying the age restriction was 50,563 participants (see Results and Table 1 for details).

**Table 1:** Participant characteristics of athletes and nonathletes (*n* = 50,653).

Demographic characteristics	Athletes (*n* = 8,447)		Nonathletes (*n* = 42,206)	
	*n*	%	*n*	%
Race				
African American/Black	440	5.2	2,164	5.1
Asian/Asian American	483	5.7	3,976	9.5
Latinae/Hispanic	369	4.4	2,912	6.9
Multiracial	928	11.0	4,930	11.7
White	5,828	69.3	26,075	62.0
Another race	360	4.3	2,002	4.8
Gender				
Man	3,631	43.3	13,848	33.1
Woman	4,712	56.2	27,589	65.9
Another gender identity	46	0.5	452	1.1
Age (years; range 18–25)	*m* = 20.26, *SD* = 1.48		*m* = 20.21, *SD* = 1.54	

*n*, number; %, percentage; *m*, mean; SD, standard deviation.

Because the item pertaining to athlete status is near the front half of the instrument, but demographic information is collected near the end of the instrument, the number of respondents for each demographic grouping does not add up to the athlete/nonathlete totals because of attrition and the ability to skip items per IRB protocol and ethical standards. Some categories do not add exactly to 100% because of the factor of rounding.

### 2.4 Athlete status

Athlete status during college was captured using the following question: “Have you been involved in the following kinds of student groups during college?” Participants selected “Yes” or “No” for several options, including the following: “Sports—Intercollegiate or Varsity (e.g., NCAA Hockey, varsity soccer)”. Participants who selected “Yes” for this question were classified as college varsity (intercollegiate) athletes, whereas participants who selected “No” for this question were classified as nonathletes.

### 2.5 Data analysis

We conducted independent t-tests and Cohen's d effect sizes to compare each SRLS subscale, the SRLS omnibus, and the LSE scale in college athletes vs. nonathletes (aim 1). We then conducted two rounds of hierarchical multiple linear regression analyses for each leadership outcome measure (SRLS subscales, SRLS omnibus, and LSE) to determine whether intercollegiate athlete involvement predicted growth in leadership capacity (SRLS) and leader self-efficacy (LSE) while controlling for other variables. All regression models included demographic variables (block 1) and reflective precollege scores (block 2) for the respective model outcomes. We included race, gender, and age because of past results that pointed to different levels of SRLS and LSE (33) and empirical research that highlighted varying and sometimes contrasting influences of collegiate experiences dependent on social identities (25, 40). Quasi pretest items and scales were used to control for precollege levels of dependent variables. Doing so allows researchers to better understand the impact of respondent experiences in college, which can reflect perceived growth (34). The first set of regression models included demographic variables, precollege scores for the respective outcome measures, and athlete status.

For the second set of hierarchical multiple linear regression models, we added a block of leadership high-impact practices. MSL researchers have repeatedly identified several key environmental predictors for leadership-related constructs (25, 33). Sociocultural conversations (i.e., discussions about and across difference), involvement in organizations on and off campus, engagement in community service, and mentoring relationships are considered leadership high-impact practices because of their positive relationships with leadership capacity and efficacy (25, 33). Because athletes may be involved in many of the above as part of their athletic experience (e.g., athletics as a form of collegiate involvement, volunteering as a team, mentoring from coaches, and meaningful conversations within diverse teams), we conducted models that both included and excluded these practices. Because all categorical variables in the regression models are effects-coded and continuous variables are standardized, unstandardized coefficients can be used to interpret effect size with categorical variables as adjusted Cohen's *d* and continuous variables as Cohen's *d* (41). A total of eight t-tests and 16 regression models (eight each for the models with and without the block of leadership high-impact practices) were conducted using IBM SPSS Statistics for Windows, version 29 (IBM Corp., Armonk, NY, USA).

## 3 Results

A total of 50,653 individuals aged 18–25 participated in this study using the 2018 MSL survey distribution, including 8,447 college varsity athletes and 42,206 nonathletes (Table 1). The SRLS subscales and the LSE scale exhibited very reliable internal consistency (Cronbach's *a* range = 0.81 to 0.90) and robust validity (RMSEA range = 0.023 to 0.049; SRMR range = 0.021 to 0.041; CFI range = 0.989 to 0.998; TLI range = 0.981 to 0.997) (42–44). Statistical assumptions were assessed and deemed appropriate for the analyses conducted.

### 3.1 Aim 1: leadership capacity and self-efficacy in college athletes vs. nonathletes

There were mixed results for the parameters of relative leadership capacity and self-efficacy for athletes compared with those for nonathletes (Table 2). Athletes reported higher levels of self-awareness and LSE compared with nonathletes. In contrast, athletes had lower levels of integrity and open-mindedness compared with nonathletes. There were no statistically significant differences in terms of personal responsibility, collaboration, civic engagement, and the overall SRLS (omnibus). All Cohen's d effect sizes were trivial (i.e., less than 0.2) (45).

**Table 2:** Leadership outcomes in athletes compared with nonathletes without controlling for other factors.

Outcome	Group	*n*	Mean (SD)	*df*	*t*	*p*	Cohen's *d*
Self-awareness	Athlete	8,314	4.03 (.63)	12,074	9.34	<0.001	0.110
	Nonathlete	41,551	3.96 (.65)				
Integrity	Athlete	8,317	4.20 (.61)	49,919	−2.11	0.035	−0.025
	Nonathlete	41,604	4.22 (.58)				
Personal responsibility	Athlete	8,320	4.36 (.58)	11,207	−1.73	0.084	−0.022
	Nonathlete	41,659	4.38 (.52)				
Collaboration	Athlete	8,306	4.17 (.58)	11,500	1.89	0.058	0.024
	Nonathlete	41,609	4.16 (.55)				
Open-mindedness	Athlete	8,311	4.18 (.58)	11,391	−5.33	<0.001	−0.067
	Nonathlete	41,658	4.22 (.54)				
Civic engagement	Athlete	8,316	3.93 (.69)	12,077	1.71	0.087	0.020
	Nonathlete	41,645	3.91 (.71)				
SRLS omnibus	Athlete	8,047	4.15 (.52)	11,095	1.74	0.082	0.022
	Nonathlete	40,270	4.14 (.49)				
LSE	Athlete	8,352	3.18 (.64)	12,392	13.79	<0.001	0.159
	Nonathlete	41,776	3.07 (.68)				

When tests indicate significant F statistics for Levene's test for equality of variances, Welch's t-test values are reported. *P*-values reported are for a two-tailed *t*-test. Cohen's d values compared athletes with nonathletes; thus, positive values indicate that athletes scored higher, whereas negative values indicate that nonathletes scored higher.

### 3.2 Aim 2: leadership capacity and self-efficacy in college athletes vs. nonathletes

For the first set of regression models that did not include leadership high-impact practices, the models explained variances in leadership (SRLS and LSE) outcomes ranging from 12.5% to 29.1% (Table 3). However, athlete status explained only approximately 0.1% or less of the variance for all outcomes, a trivial percentage that underscores its minimal practical impact. When holding constant for demographics and quasi pretest scores, college varsity athletic participation predicted trivially greater perceived growth in self-awareness, civic engagement, and LSE. In contrast, athlete status was associated with trivially less perceived growth in integrity, personal responsibility, and collaboration when controlling for precollege scores. Greater perceived growth is illustrated by positive beta (β) coefficients (Table 3), whereas less perceived growth is illustrated by negative beta (β) coefficients (Table 3). For example, in models without high-impact practices, athlete status was a significant negative predictor for integrity (β = −0.036, *p* < .001), indicating that athletes reported slightly less growth in this area compared with nonathletes.

**Table 3:** Hierarchical multiple linear regression models including demographics, precollege outcome score, and athlete status.

Predictor variables	Self-awareness		Integrity		Personal responsibility		Collaboration		Open-mindedness		Civic engagement		SRLS omnibus		Leader self- efficacy	
	β	*p*	β	*p*	β	*p*	β	*p*	β	*p*	β	*p*	β	*p*	β	*p*
Block: Demographics																
Race																
African American/Black	0.077	<0.001	0.028	0.070	0.026	0.095	0.031	0.049	−0.004	0.794	0.046	0.003	0.045	0.003	0.046	0.002
Asian/Asian American	−0.115	<0.001	−0.075	<0.001	−0.116	<0.001	−0.066	<0.001	−0.080	<0.001	−0.034	0.006	−0.090	<0.001	−0.088	<0.001
Latinae/Hispanic	0.031	0.036	0.029	0.040	0.075	<0.001	0.053	<0.001	0.077	<0.001	0.010	0.492	0.041	0.003	0.031	0.022
Multiracial	0.080	<0.001	0.098	<0.001	0.117	<0.001	0.081	<0.001	0.084	<0.001	−0.005	0.666	0.083	<0.001	0.094	<0.001
White	0.115	<0.001	0.114	<0.001	0.141	<0.001	0.098	<0.001	0.074	<0.001	0.010	0.150	0.106	<0.001	0.120	<0.001
Another Race	−0.189	<0.001	−0.194	<0.001	−0.244	<0.001	−0.196	<0.001	−0.151	<0.001	−0.026	0.104	−0.186	<0.001	−0.202	<0.001
Gender																
Man	0.056	<0.001	−0.048	<0.001	−0.044	0.002	−0.060	<0.001	−0.063	<0.001	−0.145	<0.001	−0.079	<0.001	0.044	0.001
Woman	0.180	<0.001	0.102	<0.001	0.164	<0.001	0.134	<0.001	0.142	<0.001	0.104	<0.001	0.140	<0.001	0.109	<0.001
Another Gender	−0.236	<0.001	−0.055	0.043	−0.120	<0.001	−0.074	0.006	−0.079	0.004	0.041	0.128	−0.061	0.020	−0.153	<0.001
Age	0.120	<0.001	0.070	<0.001	0.058	<0.001	0.082	<0.001	0.075	<0.001	0.075	<0.001	0.116	<0.001	0.119	<0.001
*R*^2^	0.018		0.019		0.031		0.019		0.018		0.027		0.026		0.028	
Block: precollege outcome																
Precollege Score on Respective Outcome	0.327	<0.001	0.412	<0.001	0.396	<0.001	0.432	<0.001	0.379	<0.001	0.439	<0.001	0.501	<0.001	0.514	<0.001
*R*^2^ Change	0.106		0.177		0.156		0.185		0.143		0.189		0.244		0.264	
Block: athletes																
Athlete	0.069	<0.001	−0.036	<0.001	−0.023	0.035	−0.025	0.020	−0.021	0.057	0.033	0.002	−0.002	0.872	0.042	<0.001
*R*^2^ Change	0.001		0.000		0.000		0.000		0.000		0.000		0.000		0.000	
Total *R*^2^	0.125		0.196		0.187		0.204		0.161		0.216		0.271		0.292	
Adjusted *R*^2^	0.125		0.195		0.187		0.204		0.161		0.216		0.271		0.291	
F	702	<0.001	1,198	<0.001	1,136	<0.001	1,267	<0.001	949	<0.001	1,360	<0.001	1,756	<0.001	2,028	<0.001

For the second set of regression models in which leadership high-impact practices were included, the models explained 24.4% to 41.1% of the variance in leadership capacity and self-efficacy (Table 4). Athlete status was associated with less perceived growth in all the SRLS subscales, except self-awareness and LSE, in which there was no relationship with athlete status. Athlete status again explained only approximately 0.1% or less of the variance for the second set of regression models in which leadership high-impact practices were included, again underscoring the trivial impact of collegiate athletic participation on the assessed leadership outcomes. Leadership high-impact practices appear more prevalent in athletes than nonathletes, except for sociocultural conversations (Table 5).

**Table 4:** Hierarchical multiple linear regression models including demographics, precollege outcome score, leadership high-impact practices, and athlete status. Note the addition of leadership high-impact practices to this model.

Predictor variables	Self-awareness		Integrity		Personal responsibility		Collaboration		Open-mindedness		Civic engagement		SRLS omnibus		Leader self-efficacy	
	β	*p*	β	*p*	β	*p*	β	*p*	β	*p*	β	*p*	β	*p*	β	*p*
Block: demographics																
Race																
African American/Black	0.056	<0.001	0.010	0.529	0.014	0.353	0.011	0.468	−0.032	0.035	0.025	0.077	0.023	0.109	0.033	0.022
Asian/Asian American	−0.092	<0.001	−0.059	<0.001	−0.099	<0.001	−0.048	<0.001	−0.050	<0.001	−0.025	0.029	−0.074	<0.001	−0.089	<0.001
Latinae/Hispanic	0.042	0.003	0.039	0.004	0.087	<0.001	0.069	<0.001	0.086	<0.001	0.030	0.021	0.057	<0.001	0.047	<0.001
Multiracial	0.051	<0.001	0.068	<0.001	0.088	<0.001	0.051	<0.001	0.053	<0.001	−0.035	<0.001	0.052	<0.001	0.078	<0.001
White	0.095	<0.001	0.097	<0.001	0.121	<0.001	0.081	<0.001	0.059	<0.001	−0.012	0.070	0.086	<0.001	0.108	<0.001
Another race	−0.152	<0.001	−0.155	<0.001	−0.211	<0.001	−0.164	<0.001	−0.116	<0.001	0.017	0.264	−0.144	<0.001	−0.177	<0.001
Gender																
Man	0.108	<0.001	−0.001	0.952	−0.002	0.891	−0.006	0.635	−0.012	0.399	−0.089	<0.001	−0.017	0.174	0.097	<0.001
Woman	0.142	<0.001	0.069	<0.001	0.134	<0.001	0.102	<0.001	0.110	<0.001	0.059	<0.001	0.104	<0.001	0.076	<0.001
Another gender	−0.250	<0.001	−0.068	0.008	−0.133	<0.001	−0.096	<0.001	−0.098	<0.001	0.030	0.211	−0.087	<0.001	−0.173	<0.001
Age	0.078	<0.001	0.034	<0.001	0.029	<0.001	0.044	<0.001	0.038	<0.001	0.006	0.091	0.061	<0.001	0.077	<0.001
*R*^2^	0.018		0.018		0.031		0.019		0.018		0.027		0.025		0.027	
Block: precollege outcome																
Precollege score on respective outcome	0.325	<0.001	0.386	<0.001	0.372	<0.001	0.369	<0.001	0.313	<0.001	0.331	<0.001	0.422	<0.001	0.443	<0.001
*R*^2^ change	0.105		0.175		0.155		0.186		0.143		0.189		0.243		0.266	
Block: leadership high-impact practices																
Sociocultural conversations	0.237	<0.001	0.218	<0.001	0.196	<0.001	0.225	<0.001	0.287	<0.001	0.212	<0.001	0.248	<0.001	0.152	<0.001
Community service	0.056	<0.001	0.053	<0.001	0.042	<0.001	0.054	<0.001	0.045	<0.001	0.177	<0.001	0.088	<0.001	0.067	<0.001
Mentorship with:																
Faculty/instructor	0.056	<0.001	0.053	<0.001	0.074	<0.001	0.061	<0.001	0.061	<0.001	0.054	<0.001	0.075	<0.001	0.037	<0.001
Academic or student affairs professional staff	0.015	0.102	0.015	0.077	0.010	0.253	0.035	<0.001	0.046	<0.001	0.063	<0.001	0.034	<0.001	0.024	0.003
Employer	0.081	<0.001	0.027	0.003	0.028	0.001	0.049	<0.001	0.021	0.015	0.035	<0.001	0.054	<0.001	0.106	<0.001
Community member	0.040	<0.001	0.051	<0.001	0.009	0.331	0.031	0.001	0.045	<0.001	0.099	<0.001	0.056	<0.001	0.040	<0.001
Parent/guardian	0.100	<0.001	0.091	<0.001	0.121	<0.001	0.078	<0.001	0.060	<0.001	0.016	0.081	0.068	<0.001	0.027	0.004
Another student	0.037	<0.001	0.012	0.202	0.033	<0.001	0.040	0.004	0.044	<0.001	0.071	<0.001	0.049	<0.001	0.028	0.001
On campus involvement	0.092	<0.001	0.095	<0.001	0.088	<0.001	0.099	<0.001	0.072	<0.001	0.120	<0.001	0.110	<0.001	0.121	<0.001
Off-campus involvement	0.040	<0.001	0.031	<0.001	0.004	0.340	0.018	<0.001	0.003	0.449	0.071	<0.001	0.037	<0.001	0.033	<0.001
*R*^2^ change	0.121		0.098		0.080		0.103		0.124		0.176		0.141		0.080	
Block: athletes																
Athlete	0.010	0.337	−0.096	<0.001	−0.072	<0.001	−0.074	<0.001	−0.072	<0.001	−0.057	<0.001	−0.068	<0.001	−0.010	0.322
*R*^2^ change	0.000		0.001		0.001		0.001		0.001		0.000		0.001		0.000	
Total *R*^2^	0.244		0.293		0.267		0.309		0.285		0.393		0.411		0.374	
Adjusted *R*^2^	0.244		0.293		0.267		0.308		0.285		0.392		0.411		0.373	
F	757	<0.001	969	<0.001	854	<0.001	1,045	<0.001	936	<0.001	1,515	<0.001	1,571	<0.001	1,395	<0.001

**Table 5:** Leadership high-impact practices. Values are reported as percentages for yes/no responses and as means (standard deviations) for Likert scale questions.

Leadership high-impact practices	Athletes	Nonathletes
Sociocultural conversations (range = 0–3)	1.68 (0.76)	1.69 (0.78)
Mentoring relationships (% indicating yes)		
w/Faculty or instructor	73%	68%
w/Academic or student affairs	53%	50%
w/Employer	41%	39%
w/Community member (not employer)	31%	27%
w/Parent or guardian	77%	72%
w/Another student	69%	65%
Involved in on-campus organization (range = 0–4)	2.69 (1.28)	2.27 (1.39)
Involved in off-campus organization (range = 0–4)	0.99 (1.30)	0.90 (1.31)

## 4 Discussion

This study investigated leadership capacity (SRLS) and efficacy (LSE) in college varsity athletes vs. nonathletes and explored the impact of college varsity sports participation on leadership capacity and efficacy. The first hypothesis—that athletes would have higher levels of leadership—was largely refuted, as athletes scored higher than nonathletes only on the leadership constructs of self-awareness and LSE, with these differences being trivial. Moreover, athletes had trivially lower levels of integrity and open-mindedness and no differences in terms of personal responsibility, collaboration, civic engagement, and the overall SRLS (omnibus) compared with their nonathlete peers. The second hypothesis was refuted, as athlete status explained only 0.1% or less of the variance in each leadership outcome. This large (*n* > 50,000) study indicates that competitive sports alone may not help people develop leadership capacity or self-efficacy, challenging widely held notions regarding sports and leadership.

### 4.1 Leadership in athletes vs. nonathletes

In stark contrast to widely held beliefs within sports, media reports (10–12), and qualitative research (24), our findings indicate that college varsity athletes do not report meaningfully greater socially responsible leadership capacity or self-efficacy than nonathletes. These results align with prior research from Huntrods et al., who found that intercollegiate team sport athletes (both collision and non-collision team sports) had significantly lower socially responsible leadership compared with nonathletes and that individual sport athletes showed no difference in their leadership development compared with nonathletes (20). While the present study did not classify athletes by sport type (e.g., team vs. individual sport, collision vs. non-collision sport), our findings—using a much larger and more recent sample—also suggest that athletes do not have meaningfully higher self-reported leadership capacity or self-efficacy.

Explanations for these results may pertain to how leadership was conceptualized and measured in both studies as well as the self-critical nature required for high-level athletic performance. Both Huntrods et al. (20) and the present study employ the SRLS, which is predicated on assumptions that leadership can be learned, is a collective process, and is targeted toward social change. Whereas these assumptions reflect those that undergird many leadership theories, there are other leadership models that emphasize directives from authority and excellent performance (27). Given coaching and captain-initiated direction and high-caliber play, sports may align better with the latter conceptualizations of leadership. Alternatively, highly competitive athletes may be more self-critical and thus may downplay their leadership capacities given their high self-expectations. Most athletes at the collegiate level are not team captains and may perceive themselves as followers, even if they possess leadership capacities. Ultimately, our findings may reflect a tension between the sports context and leadership measures and/or different comparative mindsets of high-performing athletes. Thus, studies that use measures based on other models of leadership (e.g., transformational, servant, or situational) may yield different results.

### 4.2 Leadership high-impact practices are embedded but could be optimized in sports

Across all leadership outcomes, regression coefficients for athlete status appeared to decrease when leadership high-impact practices were added to the models. This suppression effect, when including leadership high-impact practices, suggests that athletes already benefit from high-impact practices embedded in their athletic experiences, although to relatively minor degrees. High-impact leadership practices that college athletes follow—potentially, at least in part, through their sport—are critical to their leadership development, but sports participation alone may not facilitate leadership growth. These findings suggest that the minor benefits for socially responsible leadership and leader self-efficacy that are sometimes associated with athletics are likely mediated by the high-impact practices embedded within the athletic experience, rather than by sports participation itself.

Although athlete status alone had a minimal impact on leadership outcomes, particularly compared with the leadership high-impact practices block, which explained 8.0% to 17.6% of the variance in outcomes (Table 4), athletes reported more frequent participation in leadership high-impact practices, including mentoring relationships and involvement in organizations (Table 5). Athletes may experience sociocultural conversations through informal conversation with teammates who are different from them, community service as their team engages in volunteer or other service experiences, and mentorship through coaches, healthcare providers, and academic advisors. These practices may explain some of the benefits that sports provide regarding leadership development; however, athletes follow leadership high-impact practices beyond sports, and the effects associated with high-impact practices through sports are relatively small. Given that collegiate athletes have highly structured and demanding schedules, typically including around 20 h of training per week plus additional team responsibilities (e.g., film, travel, meetings, study halls, or community service), they may benefit from more intentional integration of leadership high-impact practices into athletic operations. Athletes may also develop important skills such as work ethic, discipline, and time management that translate well into their professional careers (24), lead to professional success, and are reflected in other conceptualizations of leadership.

### 4.3 Clinical implications

Coaches and healthcare providers who work with athletes can design and intentionally weave-in interventions that amplify the effect of leadership high-impact practices often encountered through competitive sports, including college varsity athletics (46). First, coaches and healthcare providers (e.g., athletic trainers, sports medicine physicians, physical therapists, and sports psychologists) have the potential to serve as meaningful mentors. These stakeholders can intentionally engage athletes in holistic development and help them uncover insights beyond sports. The depth of interpersonal relationships between athletes and their coaches and healthcare providers fosters trusting relationships that can profoundly impact athletes' lives. Second, when engaging in community service, coaches can help athletes move beyond perfunctory service and instead reflect on the impact of the service on the community and on themselves. Finally, coaches and healthcare providers can informally engage athletes in conversations about and across differences, cultivating a context inviting athletes to share their backgrounds and lenses of the world with each other while fostering an empathetic community. Weaver and Simet (47) explored ways in which professionals who work with athletes can foster these high-impact practices as well as ways in which these same professionals can impede leadership development. Ultimately, how coaches and healthcare providers engage athletes with these practices matters in terms of leadership development gains (25).

### 4.4 Strengths and limitations

There are many strengths to this study, including the exceptionally large sample size, the use of the most recent dataset available that was not impacted by the COVID-19 pandemic, and the use of multiple regression models controlling for known predictors of leadership outcomes. However, there are also limitations to consider when interpreting the findings. The leadership outcomes were limited to the SRLS and LSE, and thus the applicability of the findings to other constructs of leadership (e.g., transformational, servant, or situational) is unknown. Furthermore, the MSL employs a non-experimental design, which opens the research to other potentially unknown influences (48). Another limitation is that individuals self-reported their participation in college varsity sports, and thus neither this variable nor others were validated or cross-checked externally, as this study is anonymous; however, it is a reasonable assumption that individuals answered the questions honestly and accurately (49). The binary classification of individuals as athletes or nonathletes may suppress other potentially important considerations within sports, such as whether athletes participated in a team or individual sport or had a leadership role within their team (e.g., captain). In addition, a quasi pretest was used in lieu of a true longitudinal design because of feasibility, which can raise reasonable questions about accuracy; however, some scholars suggest that a quasi pre-posttest design may be more accurate for measuring some constructs (34, 35), although this method introduces recall bias. There was a greater proportion of women nonathletes (66%) than athletes (56%), although gender was controlled for in all regression models. Finally, given that the data were collected exclusively in the United States, the generalizability of the findings to sports teams, sociocultural settings, and institutions of higher education outside of the United States is unknown.

## 5 Conclusion

Our findings in a large sample of college varsity (intercollegiate) student athletes and nonathletes (*n* = 50,653) challenge widely held notions regarding sports and leadership, suggesting that competitive sports alone may not help people develop socially responsible leadership capacity or leader self-efficacy. While sports may provide opportunities for people to follow leadership high-impact practices, athletes may benefit from additional resources and opportunities to develop leadership skills inside and outside of sports.

## Data Availability

The datasets generated and/or analyzed during the current study are not publicly available due to confidentiality and data sharing agreements but are available from the corresponding author on reasonable written request.
